# Exploring the potential cost-effectiveness and societal burden implications of screening for fracture risk in a UK general radiography setting

**DOI:** 10.1186/s12891-024-08202-6

**Published:** 2025-02-04

**Authors:** E. Söreskog, B. Lopez, T. Bean, P. Lewis, N. Ashley, J. Da Palma Lopes, R. Meertens, A. Ratcliffe

**Affiliations:** 1 Macanda AB, Stockholm, Sweden; 2 Department of Learning, Informatics, Management and Ethics, Karolinska Institutet, Stockholm, Sweden; 3 Ibex Innovations Ltd, Sedgefield, UK; 4 Royal Cornwall Hospital, Truro, UK; 5 University of Exeter, Exeter, UK

**Keywords:** Economic evaluation, Cost-effectiveness, Osteoporosis, Screening, Bone Health

## Abstract

**Background:**

Fragility fractures lead to considerable societal costs and individual suffering. Despite the availability of cost-effective treatments for high-risk patients, a significant treatment gap exists, with many high-risk individuals remaining unidentified and untreated. The aim of this study was to explore the potential cost-effectiveness and societal impact of opportunistic screening for fracture risk with IBEX Bone Health (BH), a software solution that provides bone mineral density from wrist radiographs, in a UK general radiography setting.

**Methods:**

The study used a health economic model that compared the health outcomes and costs of screening with IBEX BH versus usual care for men and women aged 50 and older who had a forearm radiograph for any reason. The model incorporated data on fracture incidence, fracture risk reduction, mortality, quality of life, and fracture and treatment costs from published sources and Royal Cornwall Hospitals NHS Trust. Costs and health outcomes in terms of quality-adjusted life years (QALYs) were simulated over the remaining lifetime of patients. The analysis took the perspective of the National Health Service (NHS) and Personal Social Services in the UK.

**Results:**

The results showed that screening with IBEX BH was associated with a gain of 0.013 QALYs and a cost saving of £109 per patient compared with usual care, making it a dominant (cost-saving) strategy. Sensitivity analyses confirmed the robustness of the results under various assumptions. Widespread adoption of IBEX BH in the NHS was estimated to save 8,066 QALYs and £65,930,555 in healthcare costs over the lifetime of patients visiting hospitals for wrist radiographs each year.

**Conclusions:**

IBEX BH could be a cost-effective tool for early identification and prevention of fragility fractures in the UK, addressing the current challenges of low provision and access to fracture risk assessment and treatment.

**Supplementary Information:**

The online version contains supplementary material available at 10.1186/s12891-024-08202-6.

## Introduction

Fragility fractures represent a critical public health challenge, associated with significant impact on individual well-being and societal costs. In the UK alone, over 500,000 fragility fractures occurred in 2017 and the incidence is increasing [1]. One in six women will suffer a hip fracture from the age of 50, with major consequences on health and loss of independence. Fragility fractures account for 160,000 quality-adjusted life-years (QALYs) lost annually and causes 1,500 deaths every year in the UK [2]. In addition to human suffering, fragility fractures impose a major economic burden on society. The total fragility fracture-related cost was £4.6 billion in the UK in 2017 [1]. Despite the significant impact on health and quality of life and the proven cost-effectiveness of many available treatments for fracture prevention, there is a 66% treatment gap for women in the UK [3]. The majority of those at high risk of fractures remain unidentified and untreated, perpetuating the fracture cycle and declining health among the elderly population [4].

Fracture risk is mainly assessed by areal bone-mineral density (aBMD) using dual energy x-ray absorptiometry (DXA), assessment of other clinical risk factors (e.g., previous fracture, age, smoking, glucocorticoid use) and the FRAX® or QFracture® algorithms which are online tools that predicts fracture risk with or without BMD. General screening for fracture risk is currently not advocated, although the National Institute for Health and Care Excellence (NICE) recommends assessment of fracture risk in all women aged 65 years and older and men 75 years and older [5, 6]. The National Osteoporosis Guideline Group (NOGG) recommends FRAX assessment in postmenopausal women and men aged at least 50 years, with a clinical risk factor for fragility fracture, to guide further assessment of aBMD and treatment where indicated [6]. However, fracture risk is not routinely assessed in healthcare with only 17% of Fracture Liaison Services (FLS) assessing over 80% of their expected case load. The gap for the non-fracture group is likely to be much higher [7].

FLS has been shown to be an effective way of identifying patients with fragility fractures to prevent subsequent fractures and is advocated by policy makers and patient groups [6, 8]. Only around half of the National Health Services (NHS) trusts have implemented FLS, and the additional resources required to set up local FLSs has been described as a hurdle for wide-spread adoption of FLS in the UK [9]. Provision of DXA is also low in the UK (7.5 units per million), and only 32% of cases captured by an FLS had received DXA within 90 days of fracture [3, 10]. FLS focuses only on patients who have already suffered a fragility fracture, thus missing the opportunity to prevent the primary fracture. These problems could be mitigated by opportunistically screening patients for fracture risk without the need for additional appointments for the patient. A solution could be to use imaging applications that opportunistically screen for poor bone health at imaging appointments taken for other reasons.

IBEX Bone Health (IBEX BH) is medical device software based on the novel quantitative X-ray (QXR) method described previously [11–13], whereby bone density and T-score are extracted from a standard digital radiograph. The software has the advantage of being integrated with standard radiology workflow, meaning that patients attending for an X-ray for any reason can be assessed for osteoporosis at the examination site and a prediction made of the likelihood of osteoporosis at the femoral neck. This has been shown to be an effective screening tool for osteoporosis [13].

IBEX BH has been validated in phantom studies which showed non-inferiority to DXA within 95% confidence intervals (CIs), and clinical studies which demonstrate a receiver operator area under the curve (AUC) of 0.893 for a non-normal diagnosis at the femoral neck and 0.98 at the forearm [12]. A cross-sectional study (the OFFER1 study) investigated the receiver operating AUC performance of IBEX BH for prediction of osteoporosis and treatment recommendation by FRAX including aBMD following the NOGG guidelines [13]. AUCs for treatment recommendation at the ultra-distal and distal third regions of the radius were 0.95 (99% CI 0.91, 1.00) and 0.97 (99% CI 0.93, 1.00), respectively [13]. For osteoporosis prediction, the AUCs were 0.86 (99% CI 0.80, 0.91) and 0.81 (99% CI 0.75, 0.88), respectively [12]. The results demonstrate the potential of IBEX BH for opportunistic early prediction of fracture risk which is safe, causing low burden on the patient and healthcare system by integration with existing equipment and reporting systems.

## Purpose

The purpose of this study was to explore the potential impact on societal burden of illness and cost-effectiveness of opportunistic screening for osteoporosis with IBEX BH in a UK general radiography setting compared with usual care.

## Methods

### Patient population

The patient population in the model was selected to be similar to the average population visiting a hospital in the UK for a forearm DR scan relevant for osteoporosis screening. Women and men aged 50 or older, with or without a fragility fracture were included. Baseline characteristics of patients visiting hospital for forearm DR were sourced from Royal Cornwall Hospitals NHS Trust data. All wrist and DXA scans were extracted from the PACS.

From the dataset, the following data was extracted:

1. Those who had Plaster of Paris (POP) noted in the wrist DR report (extracted as off label).
2. Those who had Fracture noted in the wrist DR report.
3. Those who had a DXA scan within 12 months after wrist DR and no DXA scan after wrist DR.
4. The lowest T-score from a DXA lumbar spine or neck of femur DXA scan.
5. Whether a treatment recommendation was made on the report.

Data for whether the patient went onto treatment after recommendation, and what treatment they were prescribed was not available, so it was assumed that if they were recommended treatment they started it. In the OFFER1 data, the FRAX with aBMD NOGG recommendation was used to define the treatment cohort.

The total number of reports was from 10,492 unique patients between 2021–08-01 to 2023–07-31. 2719 (9 were excluded as they contained POP) patients had an IBEX BH compatible X-ray scan in that period. Of those, 983 had a fracture of which 192 were sent to DXA within one year and 54 were recommended treatment. Of those who did not have a fracture, 129 were sent to DXA and 33 were recommended treatment.

Cost-effectiveness results were determined from the weighted average over the prevalence of each risk factor (sex, age, T-score and wrist fracture) in the forearm DR population. In the base case analysis, we simulated costs and effects for women and men aged 50–90, with and without forearm fracture, and T-score between -1 to -6. Health effects and costs were then weighted by the proportion in the hospital data with each combination of risk factors (age, sex, forearm fracture, and if at/below each T-score). Out of the 2719 relevant observations, only 255 had T-score information which was too small to calculate weights. Therefore, we assumed that the full sample of wrist DR scan reports were representative of the population who would receive an IBEX BH compatible X-ray scan. In the subset with T-score information (4,221 patients) the mean patient age was 67 years, 80% were female, 3.6% had a wrist fracture and the mean T-score (lowest of lumbar spine and femoral neck) was -1.97 (95% CI -2.01, -1.94). All patients were assumed to be living at home (non-residential care) at baseline. For values that could not be estimated from the Royal Cornwall Hospitals NHS Trust, a dataset previously presented by Meertens et al. was used [13]. This is a sample of the over 50s population mostly from the Exeter area and was considered similar to the Royal Cornwall Hospitals NHS Trust population.

### Health economic model

**Fig. 1:**
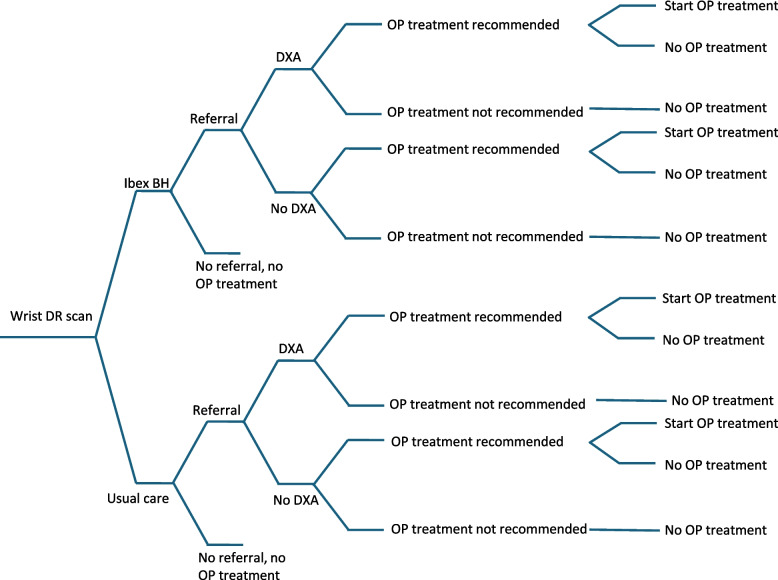
Decision tree. DR = digital radiography, OP = osteoporosis

**Fig. 2:**
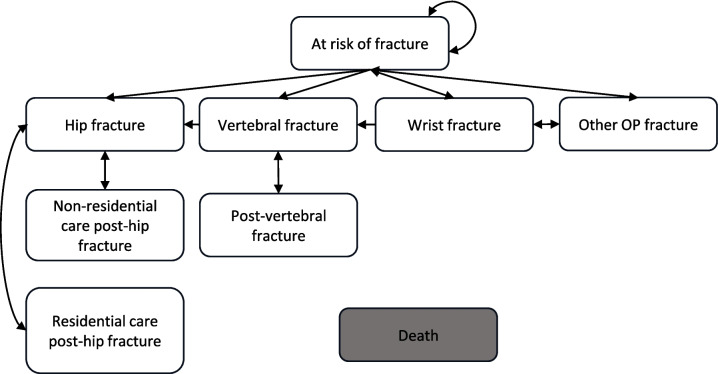
Markov model structure. All patients started in the “At risk of fracture”. Patients could stay in each health state in more than one cycle, arrows are excluded for clarity. Patients residing in residential care post-hip fracture could only transition to new hip fracture and back to residential care, not to any other state. Other OP fracture = other osteoporotic fracture

A health economic model was developed to predict the life-time consequences in terms of costs and health outcomes of opportunistic screening with IBEX BH compared with usual care. The model consisted of a decision tree, starting with forearm DR and ending with osteoporosis treatment decision (Fig. 1), followed by a Markov model (Fig. 2) with yearly cycles following the two identical patient cohorts until death or an age of 100 years. The identical cohorts entered the model at forearm DR. One cohort followed the IBEX BH pathway and the other the usual care pathway. Patients could be referred to GP for osteoporosis assessment, with or without DXA, followed by treatment decision.

The Markov model consisted of nine health states including hip fracture, vertebral fracture, forearm fracture and other fragility fracture, post-hip fracture residential care, post-hip fracture non-residential care, post-vertebral fracture, at-risk of fracture, and death. The model structure is similar to several previously published health economic models of osteoporosis interventions and a reference model created by Zethraeus et al. [14–17]. Patients started in the at risk of fracture state and, at the end of each cycle, they had a probability of remaining in the health state, incurring a hip, vertebral, forearm or other osteoporotic fracture, or dying. The Markov model had a hierarchical structure such that patients could not transition to a state with less-severe health impact in terms of quality of life and mortality than a previous state. Patients with a hip fracture transitioned to a post-hip fracture state unless they incurred a new hip fracture and could not incur a fracture at any other site. Similarly, patients with a vertebral fracture transitioned to post-vertebral state and could not transition to forearm or other osteoporotic fracture state. Patients with hip fracture were at risk of moving to residential care and would remain there for the rest of the simulation.

The model was developed in TreeAge Pro Healthcare Software (TreeAge LLC, Williamstown, MA, USA).

### Comparator usual care

Table 1 describes the base case probabilities in the decision tree. The proportion who received osteoporosis treatment in usual care was based on the Royal Cornwall data. Of all patients with forearm DR, 3.9% were recommended osteoporosis treatment and were assumed to start treatment in our model (6.1% of fractured patients and 2.7% of non-fractured patients). The probabilities of referral and DXA assessment were calibrated to reflect the proportion recommended treatment in the data.

**Table 1:** Decision tree probabilities and treatment inputs. OP = osteoporosis, HR = hazard ratio

Parameter	Value	Source and Comments
**IBEX BH strategy**		
IBEX BH sensitivity	0.93	Data from Meertens et al. [12, 13]
IBEX BH specificity	0.89	Data from Meertens et al. [12, 13]
Share of fractured patients needing OP treatment	0.28	Data from Royal Cornwall Hospitals NHS Trust Hospital [14] Based on the prevalence of treatment recommendation after DXA
Share of non-fractured patients needing OP treatment	0.18	Data from the OFFER1 trial (Meertens et al. [12, 13]). Based on the prevalence of NOGG treatment recommendation by FRAX with aBMD
Referral probability, fractured patients	0.34	Share of fractured patients needing OP treatment adjusted for IBEX BH sensitivity plus false positives (0.93*0.28 + (1–0.89)*(1–0.28))
Referral probability, non-fractured patients	0.26	Share of non-fractured patients needing OP treatment adjusted for IBEX BH sensitivity plus false positives (0.93*0.18 + (1–0.89)*(1–0.18))
Probability of receiving DXA, given they are referred for a bone health assessment	0.91	Data from Royal Cornwall Hospitals NHS Trust Hospital
Probability of starting OP treatment, fractured patients after DXA	0.77	Share of fractured patients needing OP treatment adjusted for IBEX BH sensitivity by referral probability (0.93*0.28/0.34). Note that the probability of starting OP treatment is higher than usual care owing to the fact that IBEX BH is screening patients ahead of DXA
Probability of starting OP treatment, non-fractured patients after DXA	0.64	Share of non-fractured patients needing OP treatment adjusted for IBEX BH sensitivity by referral probability (0.93*0.18/0.263)
Probability of starting OP treatment, patients without DXA, fractured and non-fractured	1	Data from Royal Cornwall Hospitals NHS Trust Hospital Data on referrals that did not end with treatment recommendation or DXA were not available. It was assumed that referred all patients who did not have DXA received treatment (9% of those referred for osteoporosis assessment)
**Usual care**		
Referral probability, fractured patients	0.20	Data from Royal Cornwall Hospitals NHS Trust Hospital Calibrated to reflect proportion recommended treatment in current care since there was no data available on referrals that did not end with treatment recommendation or DXA
Referral probability, non-fractured patients	0.08	
Probability of receiving DXA, fractured patients	0.97	
Probability of receiving DXA, non-fractured patients	0.91	
Probability of starting OP treatment, fractured patients after DXA	0.28	
Probability of starting OP treatment, non-fractured patients after DXA	0.26	
Probability of starting OP treatment, patients without DXA, fractured and non-fractured	1	
**OP treatments received (%)**		
Alendronate	0.79	Tan et al. [15], assumed to be weekly alendronate 70 mg in the model
Other oral bisphosphonates	0.12	Tan et al. [15], assumed to be weekly risedronate 35 mg in the model
Intravenous bisphosphonates	0.01	Tan et al. [15], assumed to be annual zoledronate 5 mg in the model
Denosumab	0.08	Tan et al. [15], bi-annual subcutaneous injection 60 mg
Raloxifene	0.003	Tan et al. [15], daily oral 60 mg
Teriparatide	0.003	Tan et al. [15], daily subcutaneous injection 20 µg
**Fracture risk reduction from pharmaceutical treatment (HRs vs. placebo)**		
Hip fracture	0.64	Network meta-analysis by Davis et al. [16], weighted for drug distribution [15]
Vertebral fracture	0.49	Network meta-analysis by Davis et al. [16], weighted for drug distribution [15]
Forearm fracture	0.85	Network meta-analysis by Davis et al. [16], weighted for drug distribution [15]
Other fracture	0.77	Network meta-analysis by Davis et al. [16] non-vertebral fractures, weighted for drug distribution (15)

### Intervention IBEX BH strategy

The effect of the IBEX BH strategy was modelled as an increased proportion of patients referred for osteoporosis assessment and was assumed to increase to 34.0% for fractured patients and 25.8% for non-fractured patients. Sensitivity and specificity of IBEX BH screening were based on the ROC curve provided in the OFFER1 study [13] and was chosen to match the sensitivity of FRAX (0.93) at which IBEX BH predicted treatment outcomes using NOGG guidelines with specificity of 0.89. The IBEX BH strategy increased the share of patients treated for osteoporosis (26.9% of fractured patients and 17.6% of non-fractured patients). The proportion receiving treatment in the IBEX BH strategy was varied in sensitivity analysis.

### Fracture risk and treatment efficacy

Probabilities of hip, vertebral, forearm and other osteoporotic fractures were based on age and sex specific UK general population fracture incidences. Hip and forearm fracture incidences were taken from Singer et al. [18]. Incidences of vertebral and other osteoporotic fractures were taken from Hernlund et al. [19]. Incidences were transformed to transition probabilities (1-exp(-incidence)) in the model. Probabilities were adjusted to the increased risk of the simulated patient population and potential risk reduction from osteoporosis treatment. Probabilities of the patient population versus the age and sex matched general population were adjusted to baseline aBMD T-score and forearm fracture prevalence. The relative risk of fracture per standard deviation of change in aBMD was based on a meta-analysis by Marshall et al. [20]. Reference femoral neck aBMD was based on the NHANES III survey [21]. Relative risk of fracture following forearm fracture was based on Klotzbuecher et al., which were for subsequent forearm fracture 3.3, vertebral fracture 1.7, hip fracture 1.9, and all non-vertebral fractures 2.5 applied for other fractures, based on the peri/postmenopausal population [22]. The relative risks of prior fracture were unadjusted for BMD and were therefore down adjusted by 10% [23].

Only pharmaceutical treatments were included in the model. Distribution among drugs was based on a study by Tan et al. which analysed drug utilisation of osteoporosis medications in European electronic health databases including the UK [24]. The study reported the use of oral and intravenous bisphosphonates, denosumab, teriparatide and selective oestrogen receptor modulators, which were included in our model (Table 1). Efficacy of pharmaceutical treatments was based on a meta-analysis conducted in a health technology assessment commissioned by NICE and weighted by drug distribution from Tan et al. [25]. Sensitivity analyses of treatment efficacy were conducted by up- and down-adjusting the point estimate hazard ratios from the meta-analysis by 20%. Details on fracture incidences, drug distributions, and treatment efficacy are described in the Supplementary material.

### Treatment adherence and residual effect

Adherence to osteoporosis drugs is known to be poor [26, 27]. A two-year treatment length was assumed in the base case as this reflects the average treatment duration based on UK prescription data [28]. Compliance, in terms of the extent to which a patient takes a drug according to instructions, was not specifically included in the model because fracture risk reductions from clinical trials are unadjusted for non-compliance. The fractional benefit of treatment compliance is unknown, and applying compliance in the model would underestimate fracture risk reduction. In sensitivity analyses, to explore variable persistence and compliance, treatment length was varied between one to ten years. Additionally, treatment efficacy was up- and down adjusted to test the impact of potential differences in compliance in clinical trials compared with clinical practice. Based on studies indicating that residual anti-fracture efficacy may persist for at least as long as treatment duration, efficacy was assumed to linearly decline after treatment discontinuation to zero over a period corresponding to treatment length, i.e. two years [29–32].

### Mortality

Age- and sex specific all-cause mortality rates for the general UK population were sourced from UK life tables 2020–2022 published by the Office for National Statistics [33]. Time-dependent increase in mortality following hip and vertebral fracture was taken from a study by Jönsson et al. [15]. Forearm and other osteoporotic fractures were not associated with increased mortality in the model. In agreement with previous health economic studies of osteoporotic treatments it was assumed that 30% of the excess mortality after a hip and vertebral fracture was related to the fracture event [15]. A sensitivity analysis was conducted assuming 100% excess mortality.

### Quality of life

In the “at-risk” health state, quality of life was based on EQ-5D-3L for age and sex matched general UK population from a model published by Ara et al. [34]. Hip, vertebral, forearm and other osteoporotic fractures were assumed to have an impact on quality of life in the first year of fracture event. To derive fracture state utility, the age and sex matched general population utility index was multiplied by 0.55 for the first year after hip fracture, 0.68 for vertebral fracture, and 0.83 for forearm fracture based on multipliers reported by Svedbom et al. [35]. Svedbom et al. did not present utility multipliers for other osteoporotic fractures and was assumed to be 0.79 calculated from utility loss presented by Kanis et al. and assuming that the baseline utility in patients with other osteoporotic fracture would be similar to patients with vertebral fracture in Svedbom et al. [35, 36]. Hip and vertebral fractures were assumed to also have an impact on quality of life in subsequent years (multipliers 0.86 and 0.85, respectively) based on Svedbom et al. Patients living in residential care following a hip fracture were assumed to have a quality of life weight of 0.625 from Tidermark et al. used by Davis et al. in a health technology assessment of osteoporotic drugs commissioned by NICE [25, 37].

### Fracture and treatment cost data

First-year cost of hip, vertebral, forearm and other fractures were sourced from Gutierrez et al. [38, 39]. Cost in subsequent years for hip and vertebral fractures were taken from Davis et al. [25]. Probability of discharge to residential care after hip fracture (4–34% depending on age), was based on a study by Nanjayan et al. [40]. Yearly cost of residential care within the NHS and Personal Social Service budget in the UK was assumed to be £48,998 based on Unit Costs of Health and Social Care 2022 from Personal Social Services Research Unit (PSSRU) [41]. Drug prices as of April 2024 were sourced from the British National Formulary online. Resource utilisation and the corresponding unit costs and sources are described in Supplementary material.

### Analysis

#### Cost-effectiveness

The main outcome was the incremental cost-effectiveness ratio (ICER) of the additional costs and the quality-adjusted life years (QALYs) gained from opportunistic screening with IBEX BH compared with usual care. The intervention was assumed to be cost-effective if the ICER was at or below NICE’s acceptability threshold for a technology to be an effective use of NHS resources (£20,000–30,000 per QALY gained) [42]. Deterministic one-way sensitivity analyses included probabilities in the treatment pathway (decision tree), treatment length, time horizon, discount rate, excess mortality, utility multipliers, and fracture-related costs. A sensitivity analysis was also conducted where cost-effectiveness results were weighted over the proportion of patients in the Royal Cornwall data below or at/above the age-specific intervention thresholds according to NOGG guidelines [6]. The 10-year hip and major osteoporotic fracture probabilities (based only on sex, age, T-score and prior fracture) were calculated for the patients in the Royal Cornwall data and were then compared with the intervention thresholds (Supplementary material).

The model can be used to analyse cost-effectiveness of screening at other body parts or using other screening tools, demonstrated by sensitivity analyses varying IBEX BH sensitivity and specificity (Supplementary material). Probabilistic sensitivity analysis (PSA) was conducted by simultaneously sampling from estimated probability distributions of decision tree probabilities, fracture probabilities, fracture utilities, fracture costs of IBEX BH versus usual care to obtain 1,000 sets of model input estimates. For each simulation, expected costs and QALYs were calculated for the IBEX BH strategy and the usual care strategy, respectively, and the difference between the two comparators. Acceptability curves were constructed for the pairwise comparison (shown in Supplementary material).

In the base case, costs, life years and QALYs were discounted at 3.5% annually in accordance with NICE’s reference case [42]. Costs are stated in 2024 GBP (£). The analysis took an NHS and Personal Social Services perspective and indirect costs related to e.g., productivity losses were not included.

#### Impact of opportunistic screening with IBEX BH on societal burden of osteoporosis

The model compared the burden of osteoporosis in a care pathway with IBEX BH and without it (current usual care) by employing an incidence-based bottom-up approach containing the number of patients in the target population in the UK multiplied by the corresponding disease-related consequences. Disease-related consequences included fracture costs, bed days, life years, QALYs and the indirect cost of lost QALYs caused by fractures. A hip fracture was assumed to be associated with 20.2 bed days based on 2023 data from the National Hip Fracture Database, wrist fracture 5.4 bed days and other fractures 10.6 bed days from Stevenson et al. (length of stay for other fractures based on humerus fracture data) [43, 44]. The monetary value of a lost QALY was assumed to be £20,000–£30,000 based on NICE’s willingness-to-pay threshold [42].

## Results

### Base case cost-effectiveness

Base case cost-effectiveness results show that the total costs of Screening with IBEX BH were £14,266 versus £14,375 in the usual care strategy, resulting in a cost reduction of £109 per patient (Table 2). Costs related to fractures including direct healthcare costs and nursing homes constituted the majority of total costs. Screening with IBEX BH had 0.013 more discounted QALYs and 0.012 more discounted life-years than usual care. Screening with IBEX BH was the dominant (cost-saving) strategy.

**Table 2:** Base case analysis for incremental cost-effectiveness

	**Screening with IBEX BH**	**Usual care**	**Difference**
**Cost components (£)**			
Fracture care costs	3,684	3,726	-42
Nursing home costs	10,521	10,628	-107
Intervention costs	61	21	40
Total costs	14,266	14,375	-109
**Effects**			
QALYs	9.198	9.185	0.013
Life years	11.604	11.592	0.012
**ICER (£)**			Cost-saving

Incremental total costs and incremental QALYs by age group, sex, fracture prevalence and T-score at baseline are presented in Table 3. Screening with IBEX BH lead to total cost savings in most groups, except those with the lowest fracture risk explained by higher T-score, lower age, and no fracture at baseline. Women and men with prior fracture had higher QALY gain and larger cost reductions than those without prior fracture.

**Table 3:** Incremental costs, quality-adjusted life years (QALYs) and incremental cost-effectiveness ratio (ICER) by sex, age group, fracture prevalence and T-score at baseline

	**Age 50**–**59**		**Age 60**–**69**		**Age 70**–**79**		**Age 80–90**	
**T-score**	**Fracture**	**No fracture**	**Fracture**	**No fracture**	**Fracture**	**No fracture**	**Fracture**	**No fracture**
**Incremental costs**								
Women								
-1	£34	£37	£25	£33	-£13	£14	-£56	-£5
-1.5	£28	£34	£14	£27	-£45	-£1	-£111	-£32
-2	£20	£29	-£1	£19	-£92	-£24	-£197	-£73
-2.5	£10	£24	-£23	£8	-£161	-£58	-£327	-£136
-3	-£4	£16	-£54	-£8	-£263	-£110	-£523	-£232
-3.5	-£22	£6	-£96	-£31	-£409	-£185	-£813	-£378
-4	-£48	-£8	-£155	-£62	-£616	-£294	-£1 232	-£594
Men								
-1	£35	£37	£28	£34	£7	£24	-£14	£14
-1.5	£28	£34	£18	£29	-£14	£14	-£46	-£1
-2	£19	£29	£4	£22	-£46	-£1	-£96	-£24
-2.5	£6	£23	-£17	£12	-£92	-£24	-£171	-£60
-3	-£12	£14	-£46	-£3	-£161	-£59	-£284	-£116
-3.5	-£36	£0	-£87	-£25	-£259	-£109	-£454	-£200
-4	-£70	-£18	-£143	-£55	-£399	-£182	-£703	-£326
**Incremental QALYs**								
Women								
-1	0.003	0.002	0.003	0.002	0.005	0.002	0.005	0.002
-1.5	0.004	0.002	0.005	0.002	0.007	0.003	0.007	0.003
-2	0.006	0.003	0.006	0.003	0.009	0.005	0.010	0.005
-2.5	0.008	0.004	0.008	0.004	0.012	0.006	0.013	0.007
-3	0.010	0.005	0.011	0.006	0.016	0.008	0.018	0.009
-3.5	0.013	0.007	0.014	0.008	0.021	0.011	0.024	0.013
-4	0.017	0.009	0.018	0.010	0.026	0.015	0.031	0.017
Men								
-1	0.003	0.001	0.003	0.002	0.004	0.002	0.004	0.002
-1.5	0.004	0.002	0.005	0.002	0.005	0.002	0.005	0.002
-2	0.006	0.003	0.006	0.003	0.007	0.004	0.007	0.003
-2.5	0.008	0.004	0.009	0.004	0.010	0.005	0.010	0.005
-3	0.011	0.006	0.012	0.006	0.014	0.007	0.014	0.007
-3.5	0.015	0.008	0.016	0.008	0.018	0.010	0.019	0.009
-4	0.020	0.010	0.021	0.011	0.024	0.013	0.025	0.013
**Incremental cost-effectiveness ratio (ICER)**								
Women								
-1	£10 437	£22 524	£7 537	£20 016	Cost-saving	£6 114	Cost-saving	Cost-saving
-1.5	£6 422	£15 202	£3 144	£12 151	Cost-saving	Cost-saving	Cost-saving	Cost-saving
-2	£3 447	£9 806	Cost-saving	£6 283	Cost-saving	Cost-saving	Cost-saving	Cost-saving
-2.5	£1 245	£5 843	Cost-saving	£1 880	Cost-saving	Cost-saving	Cost-saving	Cost-saving
-3	Cost-saving	£2 937	Cost-saving	Cost-saving	Cost-saving	Cost-saving	Cost-saving	Cost-saving
-3.5	Cost-saving	£791	Cost-saving	Cost-saving	Cost-saving	Cost-saving	Cost-saving	Cost-saving
-4	Cost-saving	Cost-saving	Cost-saving	Cost-saving	Cost-saving	Cost-saving	Cost-saving	Cost-saving
Men								
-1	£11 320	£25 075	£8 568	£21 546	£1 827	£13 454	Cost-saving	£8 579
-1.5	£6 669	£16 496	£3 995	£13 249	Cost-saving	£5 557	Cost-saving	Cost-saving
-2	£3 255	£10 244	£586	£7 165	Cost-saving	Cost-saving	Cost-saving	Cost-saving
-2.5	£755	£5 710	Cost-saving	£2 708	Cost-saving	Cost-saving	Cost-saving	Cost-saving
-3	Cost-saving	£2 435	Cost-saving	Cost-saving	Cost-saving	Cost-saving	Cost-saving	Cost-saving
-3.5	Cost-saving	£64	Cost-saving	Cost-saving	Cost-saving	Cost-saving	Cost-saving	Cost-saving
-4	Cost-saving	Cost-saving	Cost-saving	Cost-saving	Cost-saving	Cost-saving	Cost-saving	Cost-saving

### Sensitivity analysis

Deterministic sensitivity analyses were conducted around the base case scenario, shown in Table 4. Screening with IBEX BH was cost-saving vs usual care in all sensitivity analyses. In analyses with a higher share of patients in need of treatment, the share treated in the screening strategy vs usual care increased, leading to higher incremental QALYs and larger total cost-savings. Similarly, a sensitivity analysis assuming that 100% of fractured patients are in need of osteoporosis treatment resulted in higher QALY gain and additional cost-savings compared with the base case scenario. In a setting where the hospital has an FLS where 80% of those fractured are referred for osteoporosis assessment, the addition of IBEX BH leads to smaller, but positive QALY gain and cost-savings compared with base case (without FLS). Longer treatment length (3, 4, 5 and 10 years) was associated with higher QALY gain and cost-savings compared with the base case two-year treatment length. One-year treatment duration decreased the QALY gain and cost-savings compared with the base case. Assuming 20% less anti-fracture efficacy of treatment reduced the QALY gain and cost-savings as well. The QALY gain increased when mortality following fracture was unadjusted for comorbidities. A time horizon of 10 years instead of lifetime decreased the QALY gain. Decreasing sensitivity of IBEX BH decreased the proportion treated for osteoporosis in the IBEX BH strategy which consequently decreased incremental QALYs and cost savings compared with the base case setting of 0.93 (shown in Supplementary material). Decreasing specificity from base case value of 0.89 also increased incremental QALYs and cost savings, due to a higher proportion of non-osteoporotic patients identified in the IBEX BH strategy.

**Table 4:** One-way sensitivity analysis

Sensitivity analysis	Base case setting	Incremental QALYs	Incremental costs (£)	Incremental cost-effectiveness ratio (£)
Population averaged over proportions below/above intervention thresholds according to NOGG	Population averaged over sex, age-group, T-score, and prior fracture	0.011	-64	Cost-saving
Proportion of patients in need of treatment + 10%	Non-fractured 18%, fractured 28%	0.014	-122	Cost-saving
Proportion of patients in need of treatment + 20%	Non-fractured 18%, fractured 28%	0.015	-135	Cost-saving
Proportion of patients in need of treatment + 30%	Non-fractured 18%, fractured 28%	0.016	-149	Cost-saving
Proportion of fractured patients in need of treatment 100%	28%	0.030	-418	Cost-saving
Referral probability fractured patients current strategy 80% (FLS)	18% / 20% IBEX BH strategy / current strategy	0.004	-37	Cost-saving
Treatment length 1 year	2 years	0.010	-71	Cost-saving
Treatment length 3 years	2 years	0.016	-149	Cost-saving
Treatment length 4 years	2 years	0.018	-190	Cost-saving
Treatment length 5 years	2 years	0.020	-230	Cost-saving
Treatment length 10 years	2 years	0.028	-393	Cost-saving
Treatment efficacy -20%	Weighted hazard ratios from network meta-analysis (see Supplementary material)	0.011	-53	Cost-saving
Treatment efficacy + 20%	Weighted hazard ratios from network meta-analysis (see Supplementary material)	0.016	-165	Cost-saving
Excess mortality 100%	30%	0.015	-57	Cost-saving
Time horizon 10 years	Lifetime	0.005	-101	Cost-saving
Discount rate of health effects & costs 0%	3.5%	0.021	-144	Cost-saving
Discount rate of health effects & costs 5%	3.5%	0.007	-101	Cost-saving

### Probabilistic sensitivity analysis

The probabilistic sensitivity analysis showed that probability of screening with IBEX BH being cost-effective at a willingness-to-pay of £20,000 was 97% (included in Supplementary material). Screening was cost-saving in 77% of simulations. Mean QALY difference was 0.008 (min: 0.00007, max: 0.112) and mean total cost difference was £-106 (min: £-7,423, max: £68).

### Impact on societal burden

QALYs lost due to fractures was estimated to 338,766 in patients who were potentially relevant for opportunistic screening in one year in the UK (total 606,337 from 2,719 IBEX BH compatible wrist scans in Royal Cornwall 2021–08-01 to 2023–07-31 multiplied by 223 NHS trusts). The number of fractures potentially avoided by screening was 4,852 (1,436 hip, 1,216 vertebral, 259 forearm, and 1,941 other fractures) over ten years. The number of saved bed days associated with the fractures was estimated to 50,980. The number of QALYs potentially gained by screening was 8,066, with a monetary value of £161,318,846, at willingness-to-pay for a QALY of £20,000 and £241,978,269 at a willingness-to-pay of £30,000. Potential costs saved by introducing opportunistic screening in this population was estimated to £65,930,555 over the remaining lifetime of patients (on average 11.6 discounted life years in the IBEX BH strategy).

## Discussion

The objective of this study was to assess the cost-effectiveness of IBEX BH as an opportunistic screening tool for fracture risk determined from a radiograph of the distal forearm in men and women in the UK compared with current usual care. The analysis was conducted using a health economic model, consisting of a decision tree and a Markov simulation model following men and women aged 50 and older with forearm radiograph and potential subsequent osteoporosis treatment. Opportunistic screening with IBEX BH was assumed to result in a higher proportion of patients treated for osteoporosis compared with current usual care (26.9% vs. 6.1% in fractured patients, 17.6% vs. 2.7% in non-fractured patients).

Averaging cost-effectiveness results over women and men, with and without baseline fracture, age group and baseline BMD T-score, IBEX BH leads to 0.013 additional QALYs and cost reduction of £109 per patient. The analysis included additional costs of screening related to increased numbers of GP referrals and pharmaceutical treatments, but not costs related to IBEX BH software. The analysis may be updated in the future when the investment for the NHS per patient has been determined.

Sensitivity analyses showed that the results were robust when varying several parameters in the model. Results were most sensitive to treatment length and time horizon. Fracture risk and cost-effectiveness are highly dependent on age, sex, and other clinical risk factors including aBMD and fracture prevalence. Separate analyses over risk factors were conducted, showing that, incremental QALYs varied between 0.001 in patients with higher T-score (-1.0 or higher) without fracture at baseline to 0.031 in older women aged 70 + with fracture and lower T-score (-4.0 or lower). Screening with IBEX BH was cost-effective in all patient groups (combinations of risk factors) at willingness-to-pay threshold £30,000, and cost-saving in most cases.

The analysis suggests the IBEX BH strategy is cost-saving given that the assumed sensitivity and specificity and consequently treatment rates are achieved. However, the scope of this conclusion is limited as the accuracy of IBEX BH as a screening tool is still uncertain because the inputs to the model are based on a single centre non-randomised study. To mitigate this limitation, a sensitivity analysis varying the sensitivity and specificity of IBEX BH is presented showing broad cost-effectiveness (supplementary material Table 7). A superior way of assessing the cost-effectiveness of the screening intervention would be to implement the technology in a randomised controlled trial and then model based on the treatment or fracture rates for the two arms. This type of trial would increase confidence in the intervention’s cost-effectiveness. The operating point on the ROC curve provided in the OFFER1 study [13] was chosen to match the high sensitivity of FRAX (0.93) and a specificity of 0.89 in the base case analysis. While a higher proportion treated leads to additional cost-savings and QALYs gained, it is desirable to not overwhelm down-stream services like DXA, which is already troubled with long waiting times. At lower sensitivity, IBEX BH strategy was associated with a lower QALY gain than the base case and smaller cost-savings (shown in Supplementary material) in terms of avoided fracture costs but with lower intervention costs inflicted on healthcare.

The model simulated a heterogenous patient population which is representative of individuals undergoing wrist X-ray. IBEX BH is more likely to identify patients at higher risk of forearm fractures (relative risk (RR) 1.7 95% CI [1.4,2.0]) than other fracture sites (RR 1.4 95% CI [1.3,1.6]) [20]. However, meta-analysis [20] reports that forearm BMD is predictive of both hip (RR 1.8 95% CI [1.4,2.2]) and vertebral fracture (RR 1.7 95% CI [1.4,2.1]). Therefore, identifying patients with low BMD at the forearm should identify patients at higher risk of hip and vertebral fractures, which are associated with higher morbidity and disability than other fractures. The relationship between IBEX BH forearm BMD measures and BMD at lumbar spine and femoral neck has been demonstrated [13], also indicating that IBEX BH is predictive of fractures other than forearm. Cost-effectiveness of screening and osteoporosis treatment is, as shown in this paper and in many previous health economic analyses, dependent on prevalence of risk factors and fracture risk [15]. This is also reflected in the intervention threshold described by, for example, NOGG’s guidelines. In this analysis, the ICER in younger women and men (around 50–60 years) without prior fracture was around £20,000–25,000 meaning that screening in this group is cost-effective at a willingness-to-pay threshold of £30,000 but not £20,000. Therefore, an age-dependent threshold for opportunistic screening may be warranted. A sensitivity analysis was conducted where we averaged cost-effectiveness results on the basis of NOGG intervention thresholds and the results indicate that base case results were robust to different weighting methods.

Our findings can be compared with previous analyses of other types of fracture prevention programs in the UK. In an analysis in 2009 by the Department of Health, an estimated £290,708 over a 5-year period in NHS acute and community service was saved by introducing an FLS and treating 77% of 767 patients with hip, vertebral, wrist and humerus fractures [45]. This corresponded to a £8.5 million cost-saving on the national level over five years. McLellan et al. conducted an economic evaluation of West Glasgow FLS in 2011 in which the FLS increased treatment rates after fragility fracture to 69% from 19% vs. usual care, saving 18 fractures, 22 QALYs and £312,000 in fracture costs per 1,000 patients [46]. Turner et al. reported a cost-effectiveness analysis of a screening program in women aged 70–85 in the UK who were randomised to either usual care or screening with FRAX and potential BMD measurement. In the screening strategy, 15% received osteoporosis treatment vs. 4% in the usual care strategy. Over a 5-year period, number of QALYs was numerically but not statistically significantly higher in the screening group vs. usual care (0.0237, 95% confidence interval -0.0034 to 0.0508) and fracture costs were reduced by around £42 [47, 48]. Differences between our study and previous analyses in QALYs gained and fracture costs avoided may be explained by differences in prevalence of different clinical risk factors in patient population.

Simplifications are always necessary in health economic modelling leading to some uncertainties. Like most economic models, multiple data sources were used to populate the model. However, much of the background data to simulate fracture epidemiology, mortality, costs and resources have been used in many previously published models and accepted by governmental bodies [14, 25]. In this model, uncertainties mainly related to decision tree data inputs. The model simulated a heterogenous population in which care pathway probabilities were averaged and sensitivity and specificity were independent of risk factors (sex, age, T-score, and prior fracture). A limitation with this approach is that, in reality, referral and treatment probabilities differ among fracture risk profiles. There was a lack of data on patients who had been referred for fracture risk assessment but did not receive DXA or osteoporosis treatment. We assumed that 100% of patients who did not have DXA after wrist DR but got referred for a bone health assessment received treatment. The referral and treatment probabilities were calibrated such that the proportion treated matched the proportions in the hospital data. A limitation of this modelling approach is that increasing the specificity of IBEX BH did not lead to the expected increase in cost savings by avoiding referrals and DXA in those who did not need treatment. Another limitation is that all patients recommended treatment were assumed to start treatment, but in reality, a share may not accept it. The analysis may be updated in the future when more detailed information on treatment pathway becomes available. The drug distribution and fracture risk reduction for osteoporosis treatment was simplified and reduced to alendronate, risedronate, zoledronate, denosumab, raloxifene, and teriparatide, based on overall distribution in a large UK sample. Additional pharmaceutical treatments for osteoporosis are available in the UK but were not included in the model due to lack of data on usage. The model construct has some limitations. The hierarchical structure may, to some extent, underestimate the number of less-severe fracture types, most notably wrist fractures as they are at the bottom of the hierarchy. The cohort approach does not allow tracking of patients; consequently quality of life and cost impact of multiple fractures are not included.

An individual-state simulation approach could have addressed these uncertainties in the model construct, but such models are burdened by first-order uncertainties introducing random noise that can distort result interpretation. Another limitation relates to cost of implementation and quality of life impact of screening. Screening has been shown to have a small to moderate negative impact on quality of life in other disease areas such as cancer [49]. The impact in osteoporosis screening has not, to our knowledge, been quantified before, but could be non-negligible. Research has been made into the acceptability of opportunistic screening with IBEX BH showing that patients and the public were generally positive and accepting of the product [50]. Despite the inevitable simplifications of the model, the findings are robust as demonstrated by the extensive sensitivity analysis. The cost-effectiveness analysis is based on a published modelling framework used and validated in several previous studies, and the results are in line with what could be expected based previous cost-effectiveness analyses of screening strategies in the UK.

Opportunistic screening during routine wrist radiograph could be a cost-effective instrument in addressing the osteoporosis treatment gap. Most available fracture prevention programs will only identify patients after they have suffered a fracture. At the same time, provision of prevention programs and access to DXA is low and unequally distributed. Around 25% wait longer than 6 weeks for DXA, with large variation across UK regions (up to 70% wait longer than 6 weeks in some regions) [51]. The product addresses several of current challenges, by providing early identification, integration with existing care pathways and healthcare equipment requiring no additional imaging or appointments to patients, and a clear benefit to patients at risk of suffering fragility fractures.

## Conclusion

The results from this analysis suggest that IBEX BH as a tool for opportunistic screening of fracture risk in a UK radiography setting could gain quality-adjusted life years and reduce fracture costs, offsetting the additional costs of osteoporosis treatment and making this strategy cost saving over the current strategy applied in UK hospitals today.

## Supplementary Information

Supplementary Material 1

## Data Availability

Original data analysed in this study are included in this published article, supplementary material or in articles listed in references.

## Abbreviations

AUC: Area Under the Curve
IBEX BH: IBEX Bone Health
aBMD: Areal Bone Mineral Density
BMD: Bone Mineral Density
CI: Confidence Interval
DR: Digital Radiography
DXA: Dual-energy Xray Absorptiometry
FLS: Fracture Liaison Service
ICER: Incremental Cost-Effectiveness Ratio
NHS: National Health Services
NICE: National Institute for Health and Care Excellence
NOGG: National Osteoporosis Guideline Group
OP: Osteoporosis
PACS: Picture Archiving and Communication System
POP: Plaster of Paris
QALY: Quality-adjusted life year
RR: Relative risk
