# Supplementary material for: Exploring the potential cost-effectiveness and societal burden implications of screening for fracture risk in a UK general radiography setting
Source: BMC Musculoskelet Disord. 2025 Feb 4;26:112. doi: 10.1186/s12891-024-08202-6 (PMC11792445; doi:10.1186/s12891-024-08202-6)
Supplement: Supplementary file 1 — Supplementary Material 1 [file 12891_2024_8202_MOESM1_ESM.docx]

**Model input data**

Table 1. Fracture incidence (per 100,000) in UK general population used to estimate fracture probabilities in the model

|  | **Hip fracture** | **Vertebral fracture** | **Wrist fracture** | **Other fracture** |
| --- | --- | --- | --- | --- |
| **Women** |  |  |  |  |
| Age 50–54 | 33 | 84 | 219 | 414 |
| Age 55–59 | 51 | 142 | 309 | 504 |
| Age 60–64 | 81 | 143 | 445 | 534 |
| Age 65–69 | 132 | 192 | 522 | 903 |
| Age 70–74 | 282 | 397 | 636 | 1292 |
| Age 75–79 | 619 | 602 | 775 | 2116 |
| Age 80–84 | 1236 | 777 | 696 | 3090 |
| Age 85+ | 2255 | 1061 | 780 | 5178 |
|  |  |  |  |  |
| **Men** |  |  |  |  |
| Age 50–54 | 45 | 115 | 100 | 390 |
| Age 55–59 | 59 | 102 | 110 | 800 |
| Age 60–64 | 81 | 193 | 34 | 753 |
| Age 65–69 | 107 | 169 | 62 | 966 |
| Age 70–74 | 176 | 269 | 65 | 1334 |
| Age 75–79 | 313 | 361 | 220 | 1323 |
| Age 80–84 | 623 | 433 | 53 | 3091 |
| Age 85+ | 1220 | 819 | 223 | 4699 |

Source: Hip and forearm fracture incidences taken from Singer et al. (1). Vertebral and other fracture taken from Hernlund et al. (2)

Table 2. Fracture risk reduction from pharmaceutical treatment from network meta-analysis by Davis et al. (hazard ratios)

|  | **Hip fracture** | **Vertebral fracture** | **Forearm fracture** | **Other fracture*** |
| --- | --- | --- | --- | --- |
| Alendronate | 0.64 | 0.5 | 0.82 | 0.77 |
| Risedronate | 0.66 | 0.52 | 0.78 | 0.73 |
| Zoledronate | 0.64 | 0.4 | 0.73 | 0.73 |
| Denosumab | 0.56 | 0.3 | 1.25 | 0.86 |
| Raloxifene | 0.94 | 0.61 | 1.65 | 0.9 |
| Teriparatide | 0.35 | 0.23 | 0.64 | 0.58 |
| Weighted HR | 0.64 | 0.49 | 0.85 | 0.77 |

*Based on non-vertebral fracture endpoint. Source: Davis et al. (3)

Table 3. Treatment monitoring and drug administration unit costs used in the model

| **Item** | **Cost per unit** | **Source** |
| --- | --- | --- |
| DXA scan (referral and monitoring during osteoporosis treatment) | £65 | 2023/25 NHS Payment Scheme: 2024/25 prices workbook  HRG RD50Z DXA scan (4) |
| GP visit (referral and monitoring during osteoporosis treatment) | £42 | PSSRU Unit Costs of Health and Social Care 2022 (5). General Practitioner, including direct care staff costs and qualification costs, per patient contact lasting 9.22 minutes. Uplifted to 2024/2025 prices using NHS indicative pay cost change for 2024/2025 (2.1%) (6) |
| Intravenous drug administration day case | £172 | 2023/25 NHS Payment Scheme: 2024/25 prices workbook HRG SP12Z Deliver Simple Parenteral Chemotherapy at First Attendance (4) |
| Subcutaneous drug administration | £12 | PSSRU Unit Costs of Health and Social Care 2022 (5). GP practice nurse, per patient contact lasting 15.5 minutes. Uplifted to 2024/2025 prices using NHS indicative pay cost change for 2024/2025 (2.1%) (6) |
| Oral drug administration | £0 | Oral drugs assumed to be patient administered |

Table 4. Drug and administration costs

|  | **Alendronate** | **Risedronate** | **Zoledronate** | **Denosumab** | **Raloxifene** | **Teriparatide** |
| --- | --- | --- | --- | --- | --- | --- |
| **% of drugs used^1^** | 79.0% | 11.7% | 1.1% | 7.5% | 0.3% | 0.3% |
| **Drug acquisition costs** | | | | | | |
| Dosing unit | 70 mg | 35 mg | 5 mg/100 ml | 60 mg | 60mg | 20 $\mu g$ |
| Dosing frequency | Weekly | Weekly | Annual | Biannual | Daily | Daily |
| Unit cost | £0.31^2^ | £0.41^2^ | £13.24^3^ | £183^2^ | £0.14^2^ | £8^2^ |
| Annual cost | £16 | £21 | £13.24 | £366 | £52 | £2,913 |
| Weighted annual drug cost | £52 | | | | | |
| **Drug administration cost** | | | | | | |
| Cost per administration | £0 (oral) | £0 (oral) | £172 (day case) | £24 (SC) | £0 (oral) | £0 (patient admin.) |
| Weighted annual admin cost | £3.6 | | | | | |

Sources: ^1^Tan et al. (7) ^2^British National Formulary (online, accessed April 2024) ^3^Davis et al. (3)

Table 5. Fracture related costs and utility multipliers

|  | **Costs** | | **Utility multipliers** | |
| --- | --- | --- | --- | --- |
| **Fracture site** | **First year** | **Second and subsequent years** | **First year** | **Second and subsequent years** |
| Hip fracture | £9 283^1^ | 119^1,3^ | 0.55 (0.53–0.57) | 0.82 (0.84–0.89) |
| Vertebral fracture | £4 705^2^ | £374^2,3^ | 0.68 (0.65–0.70) | 0.85 (0.82–0.87) |
| Forearm fracture | £971^2^ | £0 | 0.83 (0.82–0.84) | N/A |
| Other fracture | £1 471^2^ | £0 | 0.79 | N/A |
| **Residential care** | | | | |
| Residential care cost per annum | 48,998^4^ | | | |

Sources: ^1^Gutiérrez et al. 2011 (8), ^2^Gutiérrez et al. 2012 (9). ^3^Davis et al. (3) ^4^For cost of living in an institutional residential setting we applied the cost of local authority provided care for older people (age 65+) from the PSSRU (5). Total local authority expenditure (minus capital) was £1442 per week. In line with Davis et al., we assumed that 36% of patients self-fund their residential care based on report from the Care Quality Commission 2012 (10, 11).

Costs were inflated to 2024/2025-year prices using NHS cost inflation index (PSSRU Unit Costs of Health & Social Care 2022) (5) and NHS indicative pay cost change for 2024/2025 (2.1%) (6).

**Additional results**

**Deterministic sensitivity analyses**

Table 6. Incremental costs, QALYs and incremental cost-effectiveness ratio (ICER) by age group, T-score, and risk of major osteoporotic fracture is below or meets intervention threshold (IT) according to NOGG guidelines at baseline

|  | **Age 50**–**59** | | **Age 60**–**69** | | **Age 70**–**79** | | **Age 80–89** | |
| --- | --- | --- | --- | --- | --- | --- | --- | --- |
| **T-score** | **At/above IT** | **Below IT** | **At/above IT** | **Below IT** | **At/above IT** | **Below IT** | **At/above IT** | **Below IT** |
| **Incremental costs** | | | | | | | | |
| -1 | £34 | £56 | £25 | £31 | -£13 | £15 | -£30 | -£2 |
| -1.5 | £29 | £32 | £16 | £25 | -£45 | -£0 | -£70 | -£26 |
| -2 | £20 | £29 | £2 | £16 | -£92 | -£24 | -£132 | -£64 |
| -2.5 | £10 | £23 | -£20 | £8 | -£161 | -£58 | -£227 | -£122 |
| -3 | £3 | NA | -£36 | -£5 | -£248 | -£100 | -£346 | -£200 |
| -3.5 | -£13 | NA | -£62 | -£33 | -£273 | -£164 | -£549 | -£327 |
| -4 | -£36 | NA | -£104 | NA | -£390 | -£256 | -£825 | -£326 |
| **Incremental QALYs** | | | | | | | | |
| -1 | 0.003 | 0.063 | 0.003 | 0.002 | 0.005 | 0.003 | 0.003 | 0.003 |
| -1.5 | 0.004 | 0.003 | 0.005 | 0.003 | 0.007 | 0.004 | 0.004 | 0.004 |
| -2 | 0.006 | 0.003 | 0.006 | 0.004 | 0.009 | 0.005 | 0.006 | 0.005 |
| -2.5 | 0.007 | 0.004 | 0.008 | 0.004 | 0.012 | 0.007 | 0.009 | 0.007 |
| -3 | 0.008 | NA | 0.009 | 0.006 | 0.015 | 0.01 | 0.011 | 0.01 |
| -3.5 | 0.011 | NA | 0.012 | 0.008 | 0.016 | 0.012 | 0.016 | 0.014 |
| -4 | 0.014 | NA | 0.015 | NA | 0.02 | 0.014 | 0.021 | 0.013 |
| **Incremental cost-effectiveness ratio (ICER)** | | | | | | | | |
| -1 | £10 547 | £886 | £7 537 | £14 613 | Cost-saving | £5 744 | Cost-saving | Cost-saving |
| -1.5 | £6 719 | £12 556 | £3 483 | £9 367 | Cost-saving | Cost-saving | Cost-saving | Cost-saving |
| -2 | £3 351 | £10 020 | £306 | £4 417 | Cost-saving | Cost-saving | Cost-saving | Cost-saving |
| -2.5 | £1 345 | £5 744 | Cost-saving | £1 874 | Cost-saving | Cost-saving | Cost-saving | Cost-saving |
| -3 | £418 | NA | Cost-saving | Cost-saving | Cost-saving | Cost-saving | Cost-saving | Cost-saving |
| -3.5 | Cost-saving | NA | Cost-saving | Cost-saving | Cost-saving | Cost-saving | Cost-saving | Cost-saving |
| -4 | Cost-saving | NA | Cost-saving | NA | Cost-saving | Cost-saving | Cost-saving | Cost-saving |

Table 7. Incremental costs, QALYs and incremental cost-effectiveness ratio (ICER) by sensitivity and specificity of IBEX BH

| **BEX BH Sensitivity** | **IBEX BH Specificity** | **Incremental QALYs** | **Incremental costs (£)** |
| --- | --- | --- | --- |
| 0.50 | 0.50 | 0.005 | -42 |
| 0.50 | 0.60 | 0.004 | -44 |
| 0.50 | 0.70 | 0.004 | -46 |
| 0.50 | 0.80 | 0.004 | -48 |
| 0.50 | 0.90 | 0.003 | -50 |
| 0.50 | 1.00 | 0.003 | -51 |
| 0.60 | 0.50 | 0.006 | -57 |
| 0.60 | 0.60 | 0.005 | -58 |
| 0.60 | 0.70 | 0.005 | -60 |
| 0.60 | 0.80 | 0.005 | -62 |
| 0.60 | 0.90 | 0.005 | -64 |
| 0.60 | 1.00 | 0.004 | -66 |
| 0.70 | 0.50 | 0.007 | -71 |
| 0.70 | 0.60 | 0.006 | -73 |
| 0.70 | 0.70 | 0.006 | -74 |
| 0.70 | 0.80 | 0.006 | -76 |
| 0.70 | 0.90 | 0.006 | -78 |
| 0.70 | 1.00 | 0.005 | -80 |
| 0.80 | 0.50 | 0.008 | -85 |
| 0.80 | 0.60 | 0.008 | -87 |
| 0.80 | 0.70 | 0.007 | -89 |
| 0.80 | 0.80 | 0.007 | -90 |
| 0.80 | 0.90 | 0.007 | -92 |
| 0.80 | 1.00 | 0.006 | -94 |
| 0.90 | 0.50 | 0.009 | -99 |
| 0.90 | 0.60 | 0.009 | -101 |
| 0.90 | 0.70 | 0.008 | -103 |
| 0.90 | 0.80 | 0.013 | -103 |
| 0.90 | 0.90 | 0.008 | -106 |
| 0.90 | 1.00 | 0.007 | -108 |
| 1.00 | 0.50 | 0.010 | -114 |
| 1.00 | 0.60 | 0.010 | -115 |
| 1.00 | 0.70 | 0.009 | -117 |
| 1.00 | 0.80 | 0.009 | -119 |
| 1.00 | 0.90 | 0.009 | -121 |
| 1.00 | 1.00 | 0.008 | -122 |

**Probabilistic sensitivity analysis**

Table 8. Results from probabilistic sensitivity analysis (discounted)

|  | **Screening with IBEX BH** | **Usual care** |
| --- | --- | --- |
| **QALYs** |  |  |
| Mean | 9.164 | 9.156 |
| Standard deviation | 0.316 | 0.317 |
| 95% CI low | 9.145 | 9.136 |
| 95% CI high | 9.184 | 9.176 |
| Min | 1.488 | 1.448 |
| Max | 15.582 | 15.580 |
|  |  |  |
| **Costs (£)** |  |  |
| Mean | 14,216 | 14,322 |
| Standard deviation | 12,989 | 13,088 |
| 95% CI low | 13,411 | 13,511 |
| 95% CI high | 15,021 | 15,133 |
| Min | 678 | 664 |
| Max | 273,052 | 275,085 |


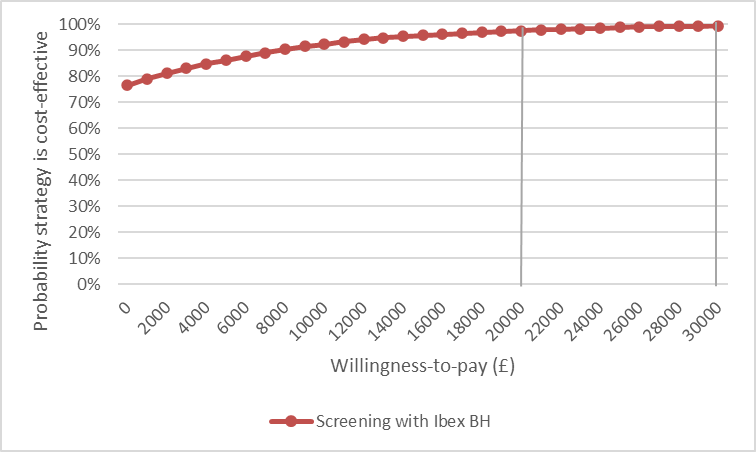


Figure 1. Cost-effectiveness acceptability curve. Vertical lines at willingness-to-pay thresholds £20,000 and £30,000, respectively

**References**

1. Singer BR, McLauchlan GJ, Robinson CM, Christie J. Epidemiology of fractures in 15,000 adults: the influence of age and gender. J Bone Joint Surg Br. 1998;80(2):243-8.

2. Hernlund E, Svedbom A, Ivergard M, Compston J, Cooper C, Stenmark J, et al. Osteoporosis in the European Union: medical management, epidemiology and economic burden. A report prepared in collaboration with the International Osteoporosis Foundation (IOF) and the European Federation of Pharmaceutical Industry Associations (EFPIA). Arch Osteoporos. 2013;8(1):136.

3. Davis S, Simpson E, Hamilton J, James MM, Rawdin A, Wong R, et al. Denosumab, raloxifene, romosozumab and teriparatide to prevent osteoporotic fragility fractures: a systematic review and economic evaluation. Health Technol Assess. 2020;24(29):1-314.

4. National Health Service England. 2023/25 NHS Payment Scheme: 2023/24 prices workbook. [Internet] Available from: <https://www.england.nhs.uk/publication/2023-25-nhs-payment-scheme/>. London, England2024.

5. Jones K, Weatherly H., Birch S, Castelli A, Chalkley M, Dargan A, et al. Unit Costs of Health and Social Care 2022 University of Kent 2022.

6. National Health Service England. 2023/25 NHS Payment Scheme (amended) Annex D: Prices and cost adjustments – 2024/25. [Internet] Available from: <https://www.england.nhs.uk/publication/2023-25-nhs-payment-scheme/> London, England2024.

7. Tan EH, Robinson DE, Jodicke AM, Mosseveld M, Bodkergaard K, Reyes C, et al. Drug utilization analysis of osteoporosis medications in seven European electronic health databases. Osteoporos Int. 2023;34(10):1771-81.

8. Gutierrez L, Roskell N, Castellsague J, Beard S, Rycroft C, Abeysinghe S, et al. Study of the incremental cost and clinical burden of hip fractures in postmenopausal women in the United Kingdom. J Med Econ. 2011;14(1):99-107.

9. Gutierrez L, Roskell N, Castellsague J, Beard S, Rycroft C, Abeysinghe S, et al. Clinical burden and incremental cost of fractures in postmenopausal women in the United Kingdom. Bone. 2012;51(3):324-31.

10. Davis S, Martyn-St James M, Sanderson J, Stevens J, Goka E, Rawdin A, et al. A systematic review and economic evaluation of bisphosphonates for the prevention of fragility fractures. Health Technol Assess. 2016;20(78):1-406.

11. The Care Quality Commission. The state of health care and adult social care in England in 2011/12. [Internet] Available from: <https://www.gov.uk/government/publications/the-state-of-health-care-and-adult-social-care-in-england-in-2011-to-2012>. London: The Stationery Office2012.
